# Clinical utility of elective paediatric flexible bronchoscopy and impact on the quality of life: protocol for a single-centre, single-blind, randomised controlled trial

**DOI:** 10.1136/bmjresp-2023-001704

**Published:** 2024-02-26

**Authors:** Rahul Thomas, Julie M Marchant, Vikas Goyal, Ian Brent Masters, Stephanie T Yerkovich, Anne B Chang

**Affiliations:** 1 Department of Respiratory and Sleep Medicine, Queensland Children's Hospital, South Brisbane, Queensland, Australia; 2 Australian Centre for Health Services Innovation, Kelvin Grove, Queensland, Australia; 3 Department of Respiratory Medicine, Queensland Children's Hospital, South Brisbane, Queensland, Australia; 4 Child Health Division, Menzies School of Health Research, Casuarina, Australia; 5 Respiratory Medicine, Australian Centre for Health Services Innovation, Kelvin Grove, Queensland, Australia

**Keywords:** Bronchoscopy, Paediatric Physician

## Abstract

**Introduction:**

Elective flexible bronchoscopy (FB) is now widely available and standard practice for a variety of indications in children with respiratory conditions. However, there are no randomised controlled trials (RCTs) that have examined its benefits (or otherwise).

Our primary aim is to determine the impact of FB on the parent-proxy quality-of-life (QoL) scores. Our secondary aims are to determine if undertaking FB leads to (a) change in management and (b) improvement of other relevant patient-reported outcome measures (PROMs). We also quantified the benefits of elective FB (using 10-point Likert scale). We hypothesised that undertaking elective FB will contribute to accurate diagnosis and therefore appropriate treatment, which will in turn improve QoL and will be deemed to be beneficial from patient and doctor perspectives.

**Methods and analysis:**

Our parallel single-centre, single-blind RCT (commenced in May 2020) has a planned sample size of 114 children (aged <18 years) recruited from respiratory clinics at Queensland Children’s Hospital, Brisbane, Australia. Children are randomised (1:1 concealed allocation) within two strata: age (≤2 vs >2 years) and indication for FB (chronic cough vs other indications) to either (a) early arm (intervention where FB undertaken within 2 weeks) or (b) delayed (control, FB undertaken at usual wait time). Our primary outcome is the difference between groups in their change in QoL at the T2 timepoint when the intervention group has had the FB and the control group has not. Our secondary outcomes are change in management, change in PROMs, adverse events and the Likert scales.

**Ethics and dissemination:**

The human research ethics committee of the Queensland Children’s Hospital granted ethical clearance (HREC/20/QCHQ/62394). Our RCT is conducted in accordance with Good Clinical Practice and the Australian legislation. Results will be disseminated through conference presentations, teaching avenues, workshops, websites and publications.

**Registration:**

Australia New Zealand Clinical Trial Registry ACTRN12620000610932.

**WHAT IS ALREADY KNOWN**

- Elective flexible bronchoscopy (FB) is now a routine procedure undertaken in children as part of the investigatory pathway when the child has respiratory symptoms/signs and/or diseases. However, the frequency of its use differs among centres which is possibly related to the current lack of high-level evidence for its use.

**WHAT THIS STUDY ADDS**

- Our study will be the first randomised controlled trial (RCT) to evaluate the clinical utility of elective FB in children and its impact on patient-related outcomes.

**HOW THIS STUDY MIGHT AFFECT RESEARCH, PRACTICE OR POLICY**

- Our study will provide the first high-level evidence in the field of paediatric FB. If our hypotheses are correct, our RCT may influence national and international clinical practice, especially in centres where FB is very commonly or uncommonly undertaken.

## Introduction

Flexible bronchoscopy (FB) involves viewing the larynx and lower airways with a flexible bronchoscope. Bronchoalveolar lavage (BAL) is usually performed at the time of FB. BAL provides various details including cellular profile and microbiology of pathogens. It is primarily used in children for diagnostic purposes and sometimes is also therapeutic.^1 2^ FB is commonly used in many paediatric respiratory centres around the world in the acute/emergency settings as well as in the non-acute (or elective settings) to help in the diagnosis and guide management in patients with respiratory symptoms, for example, chronic cough, recurrent croup and recurrent wheeze.^3^ However, the frequency of its use differs among centres, particularly in the elective settings, the focus of this study.

The publications^1 2^ on the indications, contraindications and complications of using FB in children are based on expert opinion and/or retrospective studies. Indications for undertaking FB are varied and include examination of the laryngo-tracheobronchial tree for structural and congenital airway lesions that contribute to, or the cause of, respiratory symptoms (eg, stridor/noisy breathing, persistent/recurrent wheezing, persistent radiographic abnormality, chronic cough and haemoptysis) and therapeutic indications (eg, evaluation/removal of foreign body or mucus plugs).^4^ Information gained from undertaking non-urgent FB arguably lead to attaining an accurate diagnosis (eg, diagnosis of tracheobronchomalacia in children with chronic wheeze^5^ and identification of bacterial infection).^6^ As diagnostic clarity leads to appropriate management and improvement of symptoms, the quality of life (QoL) of patients and/or their parents should improve following undertaking FB.

Currently, there are no randomised controlled trials (RCTs) examining the utility of elective FB in children with respiratory conditions. Indeed, two international thoracic societies (American Thoracic Society^7^ and European Respiratory Society)^4^ highlight the paucity of prospective studies and the absence of any RCTs examining the utility of FB in children. This is a critical knowledge gap, as FBs in children are usually undertaken using general anaesthesia with its associated risks of adverse events in addition to those from FB itself (eg, hypoxia, nausea, vomiting, headaches, confusion and unscheduled hospitalisation).^8^ Cost implications to the health system and the family are significant. For the usual day-only admissions, the mean cost is $A6071 (95% CI 5271 to 6871).^9^

Several retrospective studies have demonstrated that FB is a useful tool in children with persistent wheezing^10–12^ or ‘difficult to treat asthma’^5^ with a definitive diagnosis made in 57%–85% of cases.^10 11^ FB has been shown to provide diagnosis and change management in the evaluation of infants with stridor (33.9% of patients diagnosed with laryngomalacia and 49.5% of patients diagnosed with secondary airway lesions).^13^ FB and BAL are useful in immunocompetent children with unexplained infiltrates on chest radiographs leading to specific diagnosis in 30% of patients,^14^ as well as immunosuppressed children (diagnostic in 65.1% of patients and the antimicrobial treatment changed for 56.8%of patients).^15^ Furthermore, a specific diagnosis was made in almost 89.8% of children with congenital cardiac disease^16^ and 33% of children with unexplained recurrent and persistent pneumonia,^17^ after bronchoscopic evaluation. In another retrospective review consisting of 149 children, FB was described as a crucial diagnostic (confirming, ruling out and discovering unexpected diagnosis) and therapeutic tool.^18^

FB has also been shown to be useful in the diagnosis and management of neonates in the neonatal intensive care unit (ICU) where an abnormality was detected in 87.5% of FBs^19^ performed in neonates with noisy respiration and increased work of breathing or persistent need for respiratory assistance or persistent radiological opacities. The most common abnormalities found in neonates were tracheobronchomalacia, laryngomalacia, subglottic stenosis, choanal atresia, laryngeal cleft and tracheoesophageal fistula.^19^ FB has a high yield in children admitted to the paediatric ICU where 68% of diagnostic FBs were deemed to be abnormal, including 16% having significant extraluminal obstruction, 24% having new findings of airway anomalies and 14.5% showing endotracheal tube misplacement. Further positive microbiological results which altered or confirmed changes in patient management occurred in 46.1% of children who had a BAL during their FB in ICU.^20^ FB has also been shown to have a complementary role in the diagnosis of foreign body aspiration with a diagnostic accuracy rate of 100%, and 40% of cases were negative which stopped the operation and alleviated the need for a rigid bronchoscopy and further procedural risks.^21^

There are few prospective studies. One prospective study involving 56 indigenous children newly diagnosed with bronchiectasis (BE) found there were 25 occasions where FB and BAL altered clinical management in 23 children.^6^ A systematic review assessing the utility of FB for critically ill children (ie, non-elective FB) included 4706 patients from 27 studies (3 studies included patients above age 18 years, and none were RCTs).^22^ The authors described that using FB led to a change in medical management in 28.9% (range 21.9%–69.2%) of children.

FB is not a procedure without risk, with adverse events such as fever, hypoxia, excessive coughing, laryngospasm, epistaxis, bronchospasm and rarely pneumothorax.^8^ Therefore, it is crucial to evaluate the benefits of this procedure on clinical outcomes and patient-related outcome measures (PROMs), for example, QoL and assessing whether it leads to a change in clinical management, through the rigour of RCT. Different centres have different approaches resulting in vast differences in the number of FBs undertaken. The availability of robust evidence about the utility of FB may change clinical practice, possibly either increasing FBs utilisation in centres where FB is infrequently undertaken or reducing FBs in centres where FB is frequently used.

There is thus a need to obtain high-quality evidence to define the efficacy, benefits and adverse events associated with elective FB in the diagnosis and management of children with respiratory conditions to improve clinical outcomes for children. The ideal RCT to assess the benefits of elective FB is a randomisation to undertaking elective FB or no FB/sham FB. However, this is not ethically possible as FB has now become the standard of care.^7^

### Study objectives and hypothesis

Our primary question is as follows: among children undergoing elective FB, does this procedure lead to improvement in parents’ proxy QoL, using the age-specific Pediatric Quality of Life Inventory 4.0 (PedsQL 4.0)?^23^ We hypothesise that undertaking FB for diagnosing their child’s underlying condition leads to improved QoL in parents.

Our secondary aims are to:

1. Determine if FB leads to change in management based on a priori definitions (see below).
2. Determine if FB improves parental depression, anxiety and stress scale (DASS21)^24^ and/or the State-Trait Anxiety Inventory (STAI)^25^ scores and in the subset of children who have cough, the Parent-proxy Children’s Acute Cough-specific QoL (PAC-QoL)^26^ and cough severity scores^27^
3. Quantify the benefits (parent and doctor perspectives) of elective FB assessed by 10-point Likert scales^28^

Our secondary hypotheses is that FB will lead to:

1. Management decisions based on the a priori definitions which will lead to appropriate treatment
2. Improved PROMs (STAI,^25^ DASS21^24^ scores and/or PAC-QoL^26^ and cough severity scores,^27^ in the subset with cough)

We further hypothesised that FB will be deemed to be beneficial from both the patient and the doctor perspective.

## Methods and analysis

### Study setting and design

**Figure 1:**
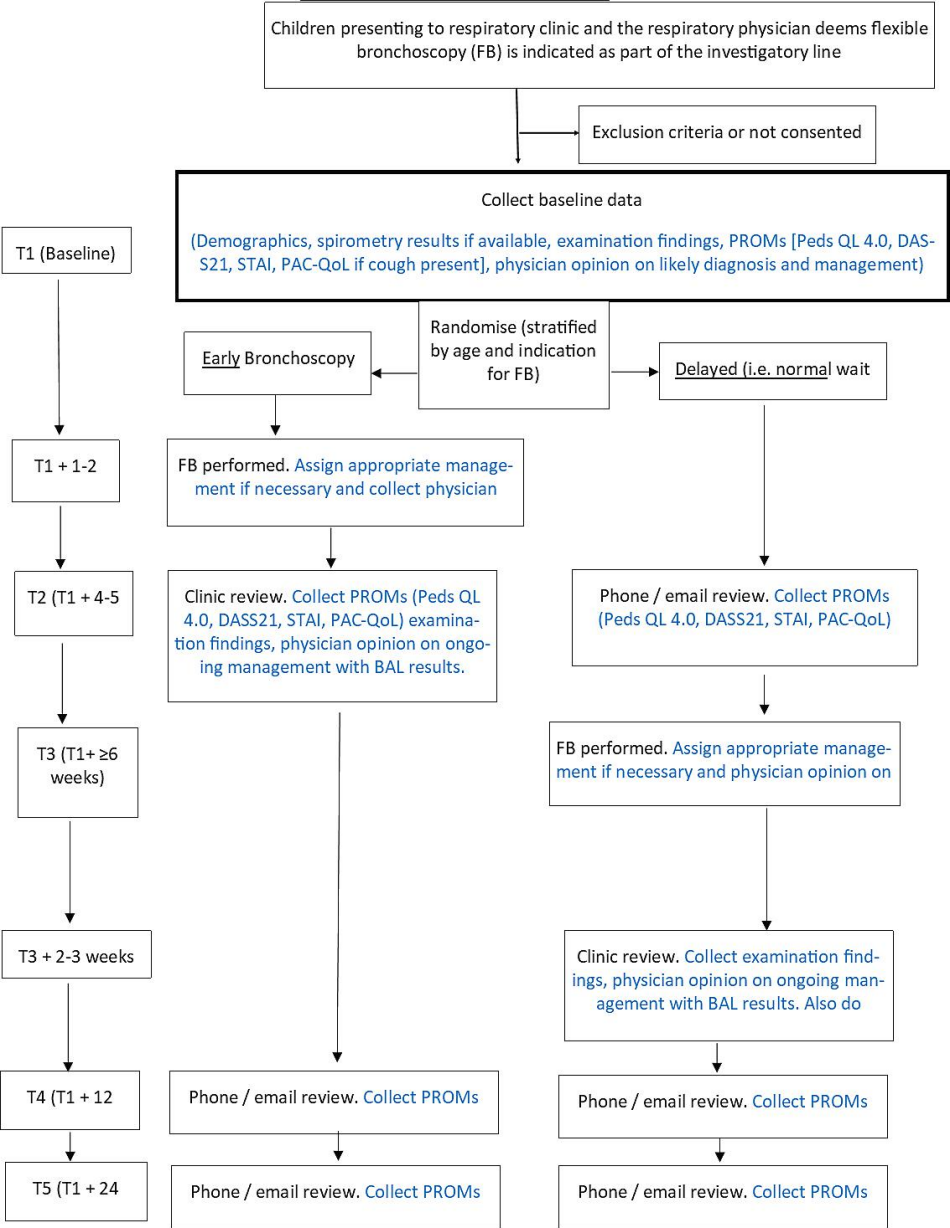
Schematic study design of the RCT. PROM, patient-related outcome measure; PedsQL 4.0,^23^ Pediatric Quality of Life Inventory 4.0; DASS21,^24^ depression, anxiety and stress scale; STAI,^25^ State-Trait Anxiety Inventory; PAC-QoL,^26^ Parent-proxy Children’s Acute Cough-specific Quality of Life; QoL, quality of life; FB, flexible bronchoscopy; BAL, bronchoalveolar lavage; T1, timepoint 1; T2, timepoint 2; T3, timepoint 3; T4, timepoint 4; T5, timepoint 5.

We are conducting a waitlist parallel, single-centre (Department of Respiratory and Sleep Medicine at the Queensland Children’s Hospital (QCH), Brisbane, Australia), single-blinded RCT with concealed allocation to determine the impact of paediatric FB on QoL and change in management. FB and BAL are done in a standard manner in the unit as per our previous studies.^29^ In our hospital, obtaining BAL is standardised.^30 31^ Further information about BAL procedures are provided in online supplemental file 1. Figure 1 summarises our study design and is in accordance with the recommendations of the Standard Protocol Items Recommendations for Interventional Trials guidelines.^32^ We recruited our first participant on 27 May 2020, and the study is ongoing with an anticipated completion of recruitment by late 2023 and complete follow-up by mid-2024.

10.1136/bmjresp-2023-001704.supp1 Supplementary data

### Patient and public involvement

Parents/guardians of patients were first involved in the preliminary study design as the study was partially based on the experience, suggestions and preferences from parents/grandparents whose children had or planned to have an elective FB. The parents/guardians of the children undergoing FB agreed with the waitlist design while being blinded to allocation. This is reflected by only one parent who has declined to participate in the study to date. We have also presented the study design to members of our parent advisory group (https://www.crelungs.org.au/cre-parent-and-community-advisory-group), and they were supportive of the study. We did not directly assess the burden of the parents’ participation in this study, but we assessed their opinion on the benefits of FB. Also, the patients were able to report any burden and could withdraw at any time. Our outcome measures included appropriate PROMs. We plan to disseminate the study outcomes to study participants during their follow-up clinical reviews through a leaflet with a summary of what our study found. We will disseminate study results to the wider community through this publication, future workshops and education sessions to health professionals, to highlight the utilisation of undertaking elective FB in children.

### Study population

Our inclusion criteria are (1) children referred to the paediatric respiratory outpatient clinic of QCH in Brisbane who require FB as deemed by the child’s primary respiratory as part of the routine clinical practice, (2) aged <18 years, (3) parents/guardian planning to remain at the study site for >6 months, (4) parents/guardians who provide informed consent able to complete the PROM questionnaires and (5) children who have not had FB previously.

Our exclusion criteria are (1) urgent FB required (eg, examination of severe stridor on child in intensive care), (2) previously enrolled or (3) limited English literacy skills.

### Recruitment

Parents/guardians of children attending QCH respiratory clinics, who are deemed to need an elective FB as part of the child’s routine investigations and who satisfy the aforementioned eligibility criteria, are approached by the primary author (RJT) either in a clinic or over the phone. The study is discussed with the parents/guardians (hereafter called parents), and a parent information sheet is provided. After informed written consent is obtained, the child is recruited, data are collected and the child is randomised into one of the two study arms as per below.

### Randomisation and allocation

Randomisation is stratified by as follows: (a) indication for FB (chronic cough or other indications (recurrent stridor, recurrent wheeze, haemoptysis, recurrent pneumonia, persistent radiographic changes, suspicion of foreign body, unexplained dyspnoea and others) and (b) age (≤2 or >2 years). The computer-generated permuted block (block size 4–6) randomisation allocation sequence (1:1 ratio) was prepared by an independent person not involved in the study. After informed consent, children are randomised to one of the two arms: (1) intervention group where the FB occurs early or (2) control group where the FB occurs within the normal waiting time. Parents of participants will not be informed of the usual wait times; thus, they are unaware of whether they are in early or ‘usual-wait’ group and hence will remain blinded. Blinding the physician is not possible.

### Overview of procedures for intervention and control groups

The outline of study procedures for both control and intervention groups is summarised in figure 1 and table 1. At enrolment (timepoint 1 (T1), baseline), both groups complete the various PROM questionnaires, that is, STAI, DASS21 and PedsQL 4.0. PAC-QoL (including cough severity scores) is also completed if chronic/recurrent cough is the indication for FB. All the PROM questionnaires are filled out by the parents/guardians. Children aged ≥5 years also complete a PedsQL 4.0 questionnaire. The intervention group (early-group) has their FB undertaken within 1–2 weeks of enrolment (followed by a phone call 48–72 hours after). At timepoint 2 (T2), children in the intervention group are seen in a follow-up clinic 2–3 weeks after their FB, and their parents repeat the PROM questionnaires, while the control group also repeat the PROM questionnaires (prior to their FB, ie the control group has not had their intervention yet at this timepoint) (see figure 1, table 1). At timepoint 3 (T3), the control group has their FB (followed by a phone call 48–72 hours after) and is clinically reviewed in a clinic within 2–3 weeks. Further PROMs are collected in both groups at timepoint 4 (T4), which is 12 weeks after baseline, and 24 weeks (6 months) after baseline as the final timepoint 5 (T5). All children receive the same standard of care.

**Table 1:** Outline of study procedures

Procedure undertaken	T1 (Baseline)	T1+1–2 weeks	T2 (T1+4–5 weeks)	T3 (T1+≥6 weeks)	T3+2–3 weeks	T4 (T1+12 weeks)	T5 (T1+24 weeks)
Screening for eligibility	**√ ∞**						
Informed consent	**√ ∞**						
Randomisation	**√ ∞**						
Medical history	**√ ∞**						
Medical chart review	**√ ∞**		**√ ∞**		**∞**		
Clinical assessment	**√ ∞**		**√**		**∞**		
PedsQL 4.0	**√ ∞**		**√ ∞**			**√ ∞**	**√ ∞**
DASS21	**√ ∞**		**√ ∞**			**√ ∞**	**√ ∞**
STAI QoL	**√ ∞**		**√ ∞**			**√ ∞**	**√ ∞**
PAC-QoL (if cough present)	**√ ∞**		**√ ∞**			**√ ∞**	**√ ∞**
Cough severity score (if cough present)	**√ ∞**		**√ ∞**		**∞**	**√ ∞**	**√ ∞**
Spirometry if able	**√ ∞**		**√**		**∞**		
FB		**√**		**∞**			
Post-FB phone call (48–72 hours post-FB)		**√**		**∞**			
BAL result review			**√**		**∞**		
Physician opinion on diagnosis and management	**√ ∞**	**√**	**√**	**∞**	**∞**		

^√^ = Early bronchoscopy group (intervention)

^∞^ = Normal-wait bronchoscopy group (control)

BAL, bronchoalveolar lavage; DASS21, depression, anxiety and stress scale; FB, flexible bronchoscopy; PAC-QoL, Parent-proxy Children’s Acute Cough-specific QoL; PedsQL 4.0, Pediatric Quality of Life 4.0; STAI QoL, State-Trait Anxiety Inventory Quality of Life; T1, timepoint 1; T2, timepoint 2; T3, timepoint 3; T4, timepoint 4; T5, timepoint 5.

### Further description of data collection

Data are collected from parent interview and from medical records. At baseline, demographic data, medical history, medications, tobacco smoke exposure, birth history and spirometry (if available) are recorded. The treating respiratory paediatrician undertaking the FB documents the child’s provisional expected FB findings and overall clinical diagnosis, indication for FB and proposed investigations and therapy before the FB at baseline (T1). Routine respiratory examination is also collected using standardised data collection sheets (eg, cough pointers; digital clubbing; chest wall deformity; ear, nose and throat; and auscultation). On the day of the FB, parents are asked to rate their child’s cough using validated score^27^ (which is repeated in the post-FB clinic follow-up appointment), if cough is present. After the FB has been undertaken, the treating physician documents the following:

1. The FB and overall clinical diagnosis
2. Whether FB leads to confirmation or exclusion of provisional diagnosis
3. If there is a new diagnosis and if confirmation of provisional FB diagnosis changed management
4. If exclusion of provisional FB diagnosis has reassured the clinician and/or family and/or changed existing management

The a priori definition of change in management (defined below) is documented after FB. The CT of the chest (if one is done concurrently, ie under the same general anaesthetic as the FB) diagnosis is also recorded, and if any management was new or changed due to the CT result is also recorded so as not to have this as a confounding variable. The physician fills in three 10-point Likert point questionnaires (1=not at all, 10=very much) regarding the FB contribution to diagnostic clarity, therapeutic management and education or counselling of parent(s) (how much did the FB contribute to the diagnostic clarity in this case, how much did the FB increase confidence in the therapeutic management of the child and how much did the FB aid in educating/counselling the parent/guardian). Complications occurring during the FB procedure are also collected including laryngospasm, bronchospasm, nasal trauma/epistaxis (for transnasal approach), pneumothorax, pulmonary haemorrhage and/or if there is hypoxia which necessitates the removal of bronchoscope for bag-mask ventilation or intubation. The SpO2 nadir will also be recorded in this instance.

Parents are again contacted by phone call 48–72 hours post-FB to check if there were any fevers in the first 24 hours post-FB and/or if anything occurred which led them to seek medical attention (seeing their family doctor or going to an emergency department). The parent also fills out three 10-point Likert point questionnaires (1=not at all, 10=very much) regarding how much the FB has reduced their concerns or worries, how likely are they to undergo FB again in the future if clinically indicated and how likely are they to recommend FB to another family who has had the same respiratory issue.

In the medical appointment at post-FB (2–3 weeks post-FB in the control and intervention groups), the parents/guardians of both groups are asked two more 10-point Likert questions:

1. How much has their child improved after bronchoscopy?
2. How much knowing the results of FB+BAL reduced their concerns or worries?

Three 10-point Likert point questionnaires are also filled in by the physician as aforementioned, but this time as regards to both the FB and BAL’s (previous questions were only regarding the FB, immediately after the FB, whereas in the clinic, the BAL results would be available as well) contribution to diagnostic clarity, therapeutic management and education or counselling of parent/guardian and if the results of FB and/or BAL have changed management at this point in time.

### Other patient-related outcome measures

Both groups will complete PROMs over four timepoints (see figure 1, table 1). The questionnaires are as follows:

1. STAI, DASS21 and PedsQL 4.0 for all patients
2. PAC-QoL (including cough severity scores) for patients with a cough

PedsQL 4.0 is a validated, generic assessment instrument that assesses the child’s and parent’s perceptions of health-related QoL.^23 33^ It has generic core scales (physical, emotional, social and school) and a total score, normalised to provide a maximum score of 100. There are separate questionnaires according to the age of the child (ages 1–12 months, 13–24 months, 2–4 years, 5–7 years, 8–12 years and 13–18 years). PedsQL 4.0 has been previously used to examine the burden of disease in chronic cough.^34 35^ PAC-QoL is a validated outcome measure to assess QoL related to childhood acute cough.^26^ There are 16 questions pertaining to cough. It has been used to examine the burden of disease in acute cough.^36^ A cough severity score^27^ is also performed along with the PAC-QoL. This score has three questions about the severity and the quality of the cough. The PAC-QoL is correlated with the cough severity scores.^26^ DASS21^24^ is a validated self-report instrument to measure the parent’s depression, anxiety and stress. STAI is a validated assessment instrument to measure anxiety in adults.^25^ There are two sets of 20 questions with one set of questions addressing the state of anxiety and the other set of questions addressing the trait of anxiety.

Each participant completes the study 6 months after the date of recruitment, after all information have been collected. All the data are documented on paper-based case report forms using standardise data collection sheets.

### Exit criteria during the study

Exit criteria are defined as:

1. If the physician decides that urgent FB is required and cannot wait on the elective list
2. If consent is withdrawn by parent
3. If there is any severe FB-related adverse event that precludes from the FB being completed

### Endpoints

The primary endpoint is a change in QoL determined by the PedsQL 4.0 between the two arms at T2 (figure 1) (prior to control group having their FB) and the baseline (T1). The secondary endpoints are as follows: (a) proportion of children whereby FB changes clinical management based on a priori factors, (b) change in DASS21 scores (T2 minus T1), (c) change in PAC-QoL scores (including cough severity scores) (T2 minus T1), (d) change in STAI scores (T2 minus T1), (e) Likert scores reflecting opinions relating to the benefit of FB (patient and doctor perspective) in clinical practice and (f) change in PedsQL 4.0, DASS21, PAC-QoL (including cough severity score) or STAI in T4 and/or T5 (to examine if there is any sustained effect by the intervention (FB)).

**Figure 2:**
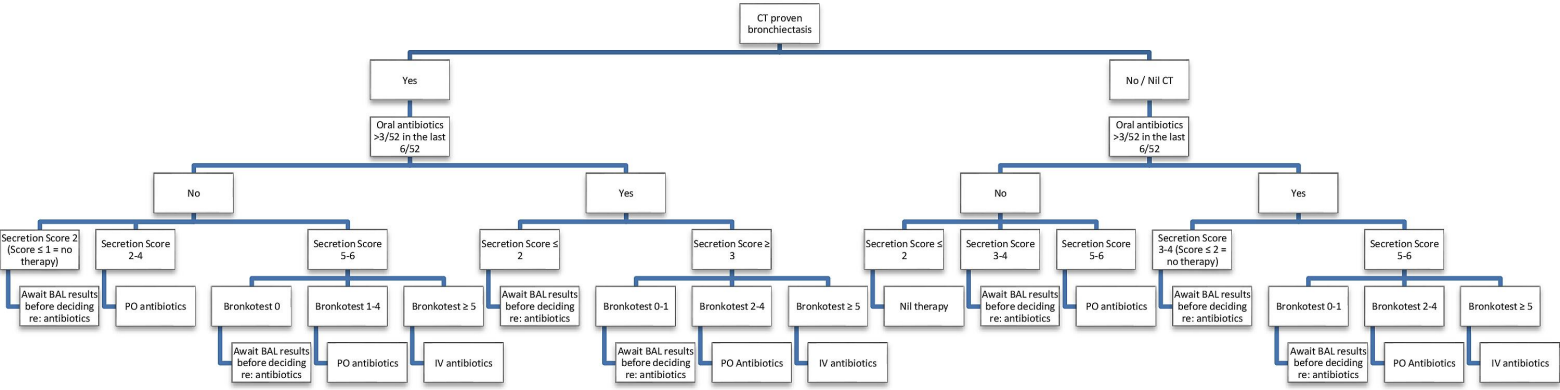
Management pathway (a priori defined management pathway based on the presence or absence (or no CT done) of bronchiectasis and on the amount of secretions and colour of secretions which are part of the bronchitis score). BAL, bronchoalveolar lavage; PO, per oral.

A priori definitions of change in management are as follows:

1. Decision to admit for intravenous antibiotics and physiotherapy directly after FB. This will be based on the secretion score^29^ and the secretion colour which is part of a validated FB bronchitis score (Bscore)^37^ (see figure 2 for details of the management pathway). The importance of this management strategy in improving clinical outcomes has been previously highlighted by research findings.^38 39^
2. Started oral (PO) antibiotics directly after FB (excluding azithromycin for CT scan-proven BE). This will also be based on the secretion score,^29^ which is part of the Bscore^37^ (see figure 2 for details of the management pathway).
3. Change in type of intravenous or PO antibiotics (or change from oral to intravenous) after BAL culture results are available(eg, anti-pseudomonal antibiotics for *Pseudomonas aeruginosa*).
4. Referred to another specialty for appropriate management changes for intervention based on findings on FB. Examples include referral to surgeons such as cardiothoracic (eg, for vascular ring), ENT (eg, subglottic lesion) and paediatric (eg, tracheo-oesophageal fistula) surgeon.

### Sample size

Our target sample size is based on finding a difference between groups of at least the minimal clinically important difference (MCID) of PedsQL 4.0 (primary outcome). With MCID of the total parent proxy of 4.5 (SD 8.25),^40^ for a study power of 90% (α=0.05), data from 48 children per group (total 96) are required. To account for a dropout of 15%, we will enrol a total of 114 children (57 children per group). For the secondary outcome of ‘change in management’, the effect size is conservatively estimated based on pilot data of a random selection of 20 FBs and using the a priori definitions aforementioned. Our pilot work found that FB led to a change in management in 14 of the 20 children (70%). The sample size of 114 provides 95% power (α=0.05) for this outcome (taking into account 15% loss).

### Data management and statistical analyses and reporting

Essential study documentation and management of all study files are in accordance with good clinical practice guidelines. Further information about data management are provided in online supplemental file 1.

Data will be reported in accordance with Consolidated Standards of Reporting Trials, and ‘intention to treat’ (ITT) analyses will be used. For PedsQL 4.0, data will not be imputed, and ITT will consist of ‘available cases’. Based on our previous RCT,^38^ the change in PedQL 4.0 is expected to be normally distributed. The difference (with 95% CI) between groups of the change in QoL will be calculated using unpaired Student’s t-test. If the groups are imbalanced at baseline, regression analyses (adjusting for baseline factors) will be used instead. For the secondary outcomes of DASS21, STAI and PAC-QoL (including cough severity scores), the same method as per PedsQL 4.0 will be used. For the outcome of ‘change in management’, χ^2^ will be used to analyse the difference in the proportion between groups, presented as odds ratio (95% CI). Likert scales regarding opinions towards benefit of FB (parent and doctor perspective) will be reported as mean (SD) or median (25th–75th percentile), based on data distribution.

### Ethics, dissemination and safety monitoring

The Human Research Ethics Committee of the Queensland Children’s Hospital granted ethical clearance (HREC/20/QCHQ/62394). Further information about dissemination and safety monitoring are provided in online supplemental file 1.

## Discussion

We are currently undertaking a parallel single-centre, single-blind, waitlist RCT with a superiority hypothesis to address the question of whether elective FB has an impact on PROMs and whether it contributes to the diagnosis and management of a child with respiratory symptoms.

### Rationale for our chosen outcome measures

Increasingly, PROMs are being used to inform healthcare decisions. Hence, our primary outcome uses a validated paediatric-specific health-related QoL (PedsQL 4.0,^23^ previously used in our multicentre RCT assessing the effectiveness of a cough algorithm for chronic cough in children)^38^ as a consumer-informed outcome measure. To date, no prospective studies have examined whether the use of paediatric elective FB has an impact on QoL. As FB usually leads to attaining an accurate diagnosis and consequently appropriate treatment, we hypothesise that QoL should improve in those who have early-FB compared with the normal-wait group (delayed-FB compared with the early-FB group).

Our secondary outcomes are DASS21,^24^ STAI^25^ and PAC-QoL^26^ (including cough severity scores—if cough present) and the ‘change in clinical management’. The 16-item PAC-QoL has been validated as a reliable outcome measure to assess QoL related to childhood cough.^26^ It has also been used to measure QoL over the duration of an acute respiratory infection with cough episode and shown to improve significantly by days 7 and 14 compared with the presentation to the emergency department at baseline with appropriate treatment.^36^ DASS21^24^ is a validated self-report instrument to measure the parent’s depression, anxiety and stress. DASS21 has been used to examine the burden of disease in chronic^41^ and acute cough.^26^ In parents of children with chronic cough, scores on all three DASS subscales reduced significantly when the children ceased coughing with appropriate treatment.^41^ The STAI has 40 items with 20 items allocated to measure the state of anxiety and 20 items allocated to measure the trait of anxiety. It is one of the most commonly used measures of general anxiety.^42^ The STAI has also been used to ascertain if listening to music while waiting for FB reduces anxiety scores in adults.^43^ In children undergoing FB for investigation of cough, there was also a significant correlation between the domains and total scores in PAC-QoL to STAI.^26^ PAC-QoL is also correlated with the cough severity scores.^27^

### Why is this study important?

The current American Thoracic Society^7^ statement on paediatric FB acknowledges the absence of any RCTs evaluating paediatric FB. Our RCT addresses this clinical research gap. Our study is the first RCT to examine the clinical use of elective FB in paediatrics for improving PROMs and/or change in clinical management. The availability of such data will guide future clinical practice worldwide. While this is the first RCT in this field, there are some potential limitations to the study. First, our RCT is a waitlist design. Since FB has become a standard of care, it is not ethically possible to undertake RCT where the control group does not have access to intervention. Second, this is a single-centre study limiting generalisability. Another potential limitation is that many patients who undergo elective FB in our centre have chronic cough as the primary indication which may affect the generalisability of the study. However, as chronic cough is the most common symptom of an underlying lung disease in children, we believe that our study would obtain results that inform future respiratory care of children.

In summary, our RCT is the first to examine the role of FB in children using the PROMs with a priori defined primary and secondary outcomes. The study results will likely inform hospital policy and will be included in future guidelines on paediatric FB in general, as well as for specific conditions such as children with chronic cough.

## Data Availability

Data are available upon reasonable request. Not applicable.
