# Supplementary material for: Clinical utility of elective paediatric flexible bronchoscopy and impact on the quality of life: protocol for a single-centre, single-blind, randomised controlled trial
Source: BMJ Open Respir Res. 2024 Feb 26;11(1):e001704. doi: 10.1136/bmjresp-2023-001704 (PMC10900573; doi:10.1136/bmjresp-2023-001704)
Supplement: Supplementary data [file bmjresp-2023-001704supp001.pdf]

<u>BAL procedure</u>

In brief, BAL was undertaken in accordance with international guidelines.[1] The first BAL lavage is taken from at least 2 lobes (the most abnormal lobes as seen on the chest CT scan or during bronchoscopy), The second lavage from a single lobe is used for differential cellular count. As per our previous studies,[2] cellular differential are performed by cytologists on cytocentrifuge slides that were prepared and stained (modified Wright stain, DiffQuik; Lab Aids; Narrabeen, NSW, Australia) (minimum of 300 cells counted). BAL is sent for total cell count (TCC), differential cellularity, quantitative bacterial culture and sensitivities and viral PCR. Any adverse events occurring during FB will be recorded such as

1. Nasal trauma and/or epistaxis,
2. Hypoxia – needing any intervention by the anaesthetist. The oxygen saturation (SpO_2) nadir will be recorded if these events occur.
    a. Bronchoscopy interrupted to facilitate bag-mask ventilation to improve oxygenation, or
    b. Requested by anaesthetist to remove bronchoscope from airway to intubate (endotracheal tube or laryngeal mask airway) patient to improve oxygenation,
3. Pneumothorax.

Any adverse events occurring after FB will be recorded such as

1. Fever (≥38° Celsius) in the first 24 hours post FB,
2. Unexpected hospitalisation or presentation to emergency department post discharge from bronchoscopy.

The anaesthetist's and post anaesthetic recovery nursing notes in the electronic medical record are reviewed.

Data Management

Individual participant files (i.e. study charts) are classified as source data and data is entered into a password-secure on-line database, Research electronic data capture (REDCap). Data is either entered directly into the study database or transcribed on paper data collection forms and later entered into the database. All entries on the database is backed up by source data, unless completed directly by participants into electronic data forms. Originals of all study source documents are retained as per institutional guidelines.

Dissemination and safety monitoring

During the study, participants may report any solicited and spontaneous adverse events at any time. All adverse events are being monitored and serious or unexpected adverse events will be reported to the HREC. The trial results, including any negative findings, will be published in an peer-reviewed journal and presented at scientific conferences, paediatric society and general practitioner meetings and other fora. Participants will be provided a written letter outlining the results of the study. The trial findings are likely to be incorporated into clinical management guidelines. Study data will be held in metadata repositories until the youngest child turns 25 years of age at the Queensland University of Technology. Deidentified study data will be made available to external parties on request and, if relevant, with the appropriate HREC approvals.

References

1. de Blic J, Midulla F, Barbato A, et al. Bronchoalveolar lavage in children. ERS Task Force on bronchoalveolar lavage in children. European Respiratory Society. *Eur Respir J* 2000;15(1):217-31. doi: 10.1183/09031936.00.15121700 [published Online First: 2000/03/04]
2. Chang AB, Faoagali J, Cox NC, et al. A bronchoscopic scoring system for airway secretions—airway cellularity and microbiological validation. *Pediatric Pulmonology* 2006;41(9):887-92. doi: 10.1002/ppul.20478
